# Is a Refundable Deductible Insurance an advantage for the insured? a mathematical approach

**DOI:** 10.1371/journal.pone.0247030

**Published:** 2021-02-17

**Authors:** M. Mercè Claramunt, Maite Mármol

**Affiliations:** Departament de Matemàtica Econòmica, Financera i Actuarial, Universitat de Barcelona, Barcelona, Spain; Shandong University of Science and Technology, CHINA

## Abstract

Most insurance policies include a deductible, so that a part of the claim is paid by the insured. In order to get full coverage of the claim, the insured has two options: purchase a Zero Deductible Insurance Policy or purchase an insurance policy with deductible together with Refundable Deductible Insurance. The objective of this paper is to analyze these two options and compare the premium paid by each. We define *dif*(*P*) as the difference between the premiums paid. This function depends on the parameters of the deductible applied, and we focus our attention on the sign of this difference and the calculation of the optimal deductible, that is, the values of the parameters of the deductible that allow us to obtain the greatest reduction in the premium.

## 1 Introduction

Currently, the car rental sector is booming and is supposed to experience an important future growth, especially in large urban centers. Specifically, the global car rental market is expected to reach a CAGR (Compound Annual Growth Rate) of 7.5 during the forecast period of 2019-2024 [1]. We could consider several reasons for this trend: a growing environmental awareness, growing traffic problems, traffic restrictions due to pollution or the explosion of car sharing.

Due to the positive evolution of this sector, companies offering insurance policies to rental companies and their customers/users have developed new strategies to complete the coverage offered and decrease the price of car hire insurance, which in some cases can represent half of the rental cost. For more information about the car rental insurance industry in Europe, see [2].

In Europe, most car rental companies offer rental insurance coverage that guarantees protection against damage, theft, and liability insurance against third parties. This is known as CDW (Collision Damage Waiver), which provides cover in the event of collision or damage, and LDW (Loss Damage Waiver), which provides cover in the event of theft or loss of use of the vehicle. These policies offer varying levels of protection against damage. In other countries, such as USA or Canada, CDW and LDW insurance must be contracted separately. Most of these insurance policies include a deductible, which in case of accident must be paid by the user to the rental company.

In order to cover the deductible, the insured has two options: He/she can purchase either an extension to a Super CDW offered by the car rental company, which is in fact a Zero Deductible Insurance, or an RDI (Refundable Deductible Insurance), which covers the cost of the deductible. In recent years, there have been online companies that, at a lower cost, cover the deductible. Then, if a claim occurs, the amount of the deductible that the car rental company has charged to the user is refunded. Thereby, if a claim occurs and the insured does not want to pay, he/she has two possibilities: purchase a Zero Deductible Insurance or a Deductible Insurance together with a Refundable Deductible Insurance.

In the actuarial literature, studies about deductibles have focused on several aspects. One of the main topics is the analysis of the problem of optimal coverage and deductible through expected utility (see [3, 4] or [5]) and stochastic dominance [6, 7]. The interaction between deductibles and bonus-malus systems and their repercussion on the efficiency of the bonus-malus system have been studied in [8–10], or [11]. Another topic is the optimal allocation of policy limits and deductibles from the viewpoint of a risk-averse policyholder [12, 13] or from the viewpoint of the insurer [14]. Although the introduction of deductibles in insurance contracts has been widely analyzed in actuarial literature, as far as we know, there are no studies concerning Refundable Deductible Insurance.

The objective of our research is to analyze the advantage the insured can have by hiring a Deductible Insurance and a Refundable Deductible Insurance, option covering the whole cost of the claim, as opposed to the alternative of directly hiring a Super CDW, that is to say a Zero Deductible Insurance policy. We measure this advantage by comparing the premium paid in these two alternatives considering different rules to share the cost of the claim between the insurer and the insured. Specifically, we worked with absolute deductible, proportional deductible, mixture of absolute deductible and proportional deductible and all-nothing deductible. For a definition of these deductibles, see [15] or [16], among others.

As a preliminary step, we will go through some concepts and facts that are useful in what follows. Notation and conventions used throughout the paper are also established. We denote by *S* the aggregate claim amount random variable (r.v.) of a given portfolio of policyholders over a year. Using the collective risk model *S* is defined as a random sum,

$$\begin{array}{c} S=\sum_{i=1}^{N}X_{i}, \end{array}$$

where $X_{i},i\in \text{I}\;\text{N}$ is a non-negative r.v. that represents the cost of the i-th claim, and *N* is a positive counting r.v. that represents the number of claims. $X_{i},i\in \text{I}\;\text{N}$ re assumed to be independent and identically distributed (i.i.d.) and also independent of *N* (see [17] or [18]). Let $A(X_{i}),i\in \text{I}\;\text{N}$ be the part of the cost paid by the insured in an insurance with deductible, and $C(X_{i}),i\in \text{I}\;\text{N}$, the part of the claim paid by the insurer. The r.v. $X_{i},i\in \text{I}\;\text{N}$ is distributed as the strictly positive r.v. *X*, with cumulative distribution function *F*_*X*_(*x*). The survival function of *X*, its expected value and variance are denoted by ${\bar{F}}_{X}\left(x\right)=1-F_{X}\left(x\right)$, *E*(*X*) and *V*(*X*), respectively. From now on, for reasons of simplicity, *A*(*X*) and *C*(*X*) are denoted as *A* and *C*.

The moments of the aggregate claim amount are easily calculated from the moments of *X* and *N*. The expected value of the aggregate claim amount *S* is:

$$\begin{array}{c} E(S)=E(N)E(X), \end{array}$$ (1)

and its variance:

$$\begin{array}{c} V\left(S\right)=E\left(N\right)V\left(X\right)+E{\left(X\right)}^{2}V\left(N\right). \end{array}$$ (2)

Without deductible, the aggregate claim amount coincides with the total cost covered by the insurer. If a deductible is applied, the total cost for the insurer is calculated with the same formula than the aggregate claim amount but including only the part of each of the claims that he/she pays (*C*) instead of considering the whole claims (*X*). From now on, in order to avoid confusions, *S* stands for the total cost paid by the insurer.

The insurer calculates the premium considering only the part of the claim that he/she pays. If the contract includes a deductible, the moments of the total cost for the insurer are obtained from (1) and (2), substituting *X* by *C*.

Let Π be the premium paid for a Zero Deductible Insurance, Π^*D*^ the premium paid for a Deductible Insurance, and Π^*R*^ the premium paid for a Refundable Deductible Insurance, that is to say an insurance policy that covers *A*. To measure the effect of the two alternatives that the insured has to get full coverage, we compare Π and Π^*D*^ + Π^*R*^. The function *dif*(*P*) measures the difference for the insured between the two options,

$$\begin{array}{c} dif\left(P\right)=\Pi -\Pi^{D}-\Pi^{R}, \end{array}$$

where *dif*(*P*) is a function that depends on *P*, the set of parameters that defines each deductible, *P* = {*p*_1_, …, *p*_*j*_}, *j* ∈ *N*, being *j* the number of parameters, with $p_{i}\in P\subset R^{+}$. For example, for an absolute deductible (the insured pays the first *a* monetary units of each claim *X*, and the insurer pays the excess over *a*), *j* = 1 and *P* = {*a*}. Then, if Π = Π^*D*^ + Π^*R*^, *dif*(*P*) equals to zero which implies that the two alternatives are indifferent for the insured. If Π < Π^*D*^ + Π^*R*^, *dif*(*P*) is negative and the insured prefers to purchase just a Zero Deductible Insurance. And lastly, if Π > Π^*D*^ + Π^*R*^, *dif*(*P*) is positive, so the insured prefers to buy a Deductible Insurance and a Refundable Deductible Insurance. This analysis depends on the type of deductible applied and the premium principle used, in other words, the mathematical method used to fix the insurance premium. In this paper, we focus our attention on two premium principles: the mean principle and the variance principle.

If we use the mean principle, the premium is calculated as the expected value of the risk plus a safety loading to this expected value,

$$\begin{array}{c} \Pi =E(S)(1+\delta ),\;\delta >0, \end{array}$$

whereas if we use the variance principle, the loading is proportional to the variance,

$$\begin{array}{c} \Pi =E(S)+\delta V(S),\;\delta >0. \end{array}$$

For more information about premium principles and their properties, see [19, 20].

If the mean principle is applied, the premium of the Deductible Insurance is

$$\begin{array}{c} \Pi^{D}=E\left(N\right)E\left(C\right)\left(1+\delta \right), \end{array}$$

the premium for the Refundable Deductible Insurance is

$$\begin{array}{c} \Pi^{R}=E\left(N\right)E\left(A\right)\left(1+\delta \right), \end{array}$$

and the premium paid for a complete insurance covering the whole claim, a Zero Deductible Insurance, is given by

$$\begin{array}{c} \Pi =E(N)E(X)(1+\delta ). \end{array}$$

Then, knowing that *E*(*X*) = *E*(*C*) + *E*(*A*), *dif*(*P*) = Π − Π^*D*^ − Π^*R*^ = 0 for any *X* and *N* and whatever deductible is used. In this case, there is no advantage from choosing one alternative or the other. Whereas, if we focus on the variance principle, it is easy to see that *dif*(*P*) is not always equal to zero, and then a deep analysis is needed in order to optimize the advantage that the insured can get.

In this framework, the paper contributes in three aspects. First we propose a theoretical framework for the market practice of hiring a Refundable Deductible Insurance policy. Second, we find a sufficient condition, fulfilled by almost all deductible types, that guarantees the advantage for the insured of the Refundable Deductible Insurance under the variance principle. And lastly, considering that the set of parameters *P* that defines each deductible is chosen by the insured, we present the optimal deductible parameters allowing the insured to obtain the maximum gain for different types of deductibles.

After this introduction, the paper is structured as follows. In Section 2, if the variance principle is applied, the expression of *dif*(*P*) valid for any deductible is obtained and the conditions that allow to maximize this function are presented. One of the main results obtained is that the commonotonicity of the parts of the claim covered by the insurer and the insured guarantees the advantage that the insured can obtain by hiring a Refundable Deductible Insurance policy. In Section 3, we develop the specific results for the different deductibles considered in this paper: absolute deductible, proportional deductible, mixture of absolute deductible and proportional deductible and all-nothing deductible. We show that the deductible parameters that maximize the difference obtained by the insured depend on the expected value and variance of the number of claims and the distribution of the individual claim amount. The paper ends with some conclusions.

## 2 Analysis and optimization of *dif*(*P*) for any deductible if the variance principle is applied

In this section the variance principle is used to calculate premiums. A general expression for *dif*(*P*) is obtained and the conditions that allow to maximize this function are presented.

Using the variance principle, the premium of the Deductible Insurance is

$$\begin{array}{c} \Pi^{D}=E\left(N\right)E\left(C\right)+\delta \left[E\left(N\right)V\left(C\right)+E{\left(C\right)}^{2}V\left(N\right)\right], \end{array}$$

whereas the premium for the Refundable Deductible Insurance is given by

$$\begin{array}{c} \Pi^{R}=E\left(N\right)E\left(A\right)+\delta \left[E\left(N\right)V\left(A\right)+E{\left(A\right)}^{2}V\left(N\right)\right], \end{array}$$

and the premium that would be paid for a complete insurance covering all *X*, a Zero Deductible Insurance, is given by

$$\begin{array}{c} \Pi =E\left(N\right)E\left(X\right)+\delta [E\left(N\right)V\left(X\right)+E{\left(X\right)}^{2}V\left(N\right)]. \end{array}$$

The random variables *A* and *C* depend not only on *X* but also on the parameters of the deductible. In order to simplify the expressions, we will not make explicit these dependencies. Then, with the variance principle,

$$\begin{array}{ccc} dif(P) & = & \delta E\left(N\right)V\left(X\right)+\delta V\left(N\right)E{\left(X\right)}^{2}-\delta E\left(N\right)\left[V\left(C\right)+V\left(A\right)\right]\\ & & -\delta V\left(N\right)[E{\left(C\right)}^{2}+E{\left(A\right)}^{2}]\\ & = & 2\delta E(N)Cov(A,C)+2\delta V(N)E(A)E(C). \end{array}$$ (3)

Taking into account that *Cov*(*A*, *C*) = *E*(*AC*) − *E*(*A*)*E*(*C*) and substituting *C* by *X* − *A*, an alternative expression for *dif*(*P*) is obtained,

$$\begin{array}{c} dif\left(P\right)=2\delta (\left[E\left(A\right)E\left(X\right)-E{\left(A\right)}^{2}\right]\left[V\left(N\right)-E\left(N\right)\right]+E\left(N\right)\left[E\left(AX\right)-E\left(A^{2}\right)\right]). \end{array}$$ (4)

We analyze this function *dif*(*P*). The first aspect is the sign of this difference. Proposition 1 establishes its positiveness under an usual condition. The second aspect studied is the calculation, if it exists, of the optimal deductible, that is, the values of the parameters of the deductible such that the insured obtains the greatest reduction in the total premium paid with the same coverage. In this section, Proposition 2, we include the first order condition for this optimization problem. Next sections are dedicated to these questions regarding the absolute deductible, the proportional deductible with maximum loss, a mixture of an absolute deductible and a proportional deductible and the all-nothing deductible.

Before Proposition 1 is presented, we introduce the definition of comonotonicity.

**Definition 1**. *Let X and Y be two non negative r.v. that represent two different risks. They are comonotonic (see* [21]) *if their bivariate cumulative distribution function*, *F*_*XY*_(*x*, *y*), satisfies *F*_*XY*_(*x*, *y*) = *min*[*F*_*X*_(*x*), *F*_*Y*_(*y*)] for all *x*, *y* ≥ 0.

From an intuitive point of view, the comonotonicity of two risks means that these risks are not able to compensate each other.

**Proposition 1**. *For a deductible such that A and C are comonotonic risks*, *dif*(*P*) > 0.

*Proof*. If two risks are comonotonic, its covariance is positive (see [22]). Then, from (3), if *A* and *C* are comonotonic, *dif*(*P*) > 0.

Then, the comonotonicity is a sufficient condition for the positiveness of *dif*(*P*). For more information on comonotonicity, see [23–25]. The r.v.’s *A* and *C* are comonotonic in almost all deductibles, see [21]. This is the case for the absolute deductible (Section 3.1), the proportional deductible with maximum loss (Section 3.2) and a mixture of an absolute deductible and a proportional deductible (Section 3.3). In Section 3.4., we analyze a type of deductible such that *A* and *C* are not comonotonic, the all-nothing deductible.

The optimal deductible problem is

$$\begin{array}{c} \max dif(P), \end{array}$$

w.r.t. the parameters {*p*_1_, …, *p*_*j*_} that define the deductible and subject to the constrains $p_{i}\in P\subset R^{+},i=1,\ldots ,j$. These constrains permit obtaining the set of feasible solutions, *D*. In all the deductibles studied in this paper, *D* turns out to be an open set, and *dif*(*P*) a continuous and differentiable function in *D*.

**Proposition 2**. *The values of the parameters of the deductible such that the insured obtains the greatest reduction in the global total premium paid with the same coverage fulfill, if they exist, the following system of equations*

$$\begin{array}{c} \frac{\partial E(AX)}{\partial p_{i}}-\frac{\partial E(A^{2})}{\partial p_{i}}=g\left(N\right)(\frac{\partial E(A)E(X)}{\partial p_{i}}-\frac{\partial E{\left(A\right)}^{2}}{\partial p_{i}}),\;i=1,...,j, \end{array}$$ (5)

being $g\left(N\right)=\frac{E(N)-V(N)}{E(N)}$. In the Poisson case, this system is reduced to

$$\begin{array}{c} \frac{\partial E(AX)}{\partial p_{i}}-\frac{\partial E(A^{2})}{\partial p_{i}}=0,\;i=1,...,j. \end{array}$$

*Proof*. The first order condition of the optimal deductible problem is

$$\begin{array}{c} \nabla dif\left(P\right)=(\frac{\partial dif(P)}{\partial p_{1}},\ldots ,\frac{\partial dif(P)}{\partial p_{j}})=0, \end{array}$$

that, taking into account the definition of *dif*(*P*) in (4), allows us to obtain (5). From (5) we see that, regarding the number of claims, only its expected value and variance affect the optimization problem and in fact, when the expected value and the variance of *N* are equal, the first order conditions are simplified. In the Poisson case, *N* ∼ *Pois*(*λ*), *E*(*N*) = *V*(*N*), so *g*(*N*) = 0, and the result presented in the proposition is obtained.

In Section 3, we apply Proposition 2 to obtain the parameters of the different deductibles that maximizes the reduction in the global total premium paid with the same coverage.

## 3 Analysis and optimization of *dif*(*P*): Specific results for different types of deductibles if the variance principle is applied

In this section we present the results obtained in the optimization of *dif*(*P*) for different types of deductibles under the variance principle. Specifically, we consider absolute deductible, proportional deductible, mixture of absolute deductible and proportional deductible and all-nothing deductible. In the first three types of deductibles, Proposition 1 is fulfilled, that is to say, *A* and *C* are comonotonic risks and therefore *dif*(*P*) is always positive. If the all-nothing deductible is applied, *A* and *C* are not comonotonic risks, thence *dif*(*P*) can be positive or negative, so, for the insured, the option of taking out a Deductible Insurance and a Refundable Deductible Insurance is not always better than hiring a Zero Insurance policy.

### 3.1 Absolute deductible

If the absolute deductible is applied, the insured pays the first *a* monetary units of each claim *X*, and the insurer pays the excess over *a*, *X* − *a*. Then, if an absolute deductible with parameter *a* ≥ 0 is applied, *A* and *C* are defined in Table 1.

**Table 1:** Definition of *A* and *C* if an absolute deductible with parameter *a* ≥ 0 is applied.

*X*	*A*	*C*
*X* < *a*	*X*	0
*X* > *a*	*a*	*X* − *a*

Following [26], we define the s-th partial moment of *X* about the origin over (0, *x*_0_) as the partial expectation of *X*^*s*^, $H_{X^{s}}\left(x_{0}\right)=\int_{0}^{x_{0}}x^{s}f\left(x\right)dx$. Hence, the expectations of *A*, *AX* and *A*^2^ are

$$\begin{array}{c} E(AX)=H_{X^{2}}\left(a\right)+aE\left(X\right)-H_{X}\left(a\right), \end{array}$$ (6)

$$\begin{array}{c} E(A^{2})=H_{X^{2}}\left(a\right)+a^{2}{\bar{F}}_{X}\left(a\right), \end{array}$$ (7)

$$\begin{array}{c} E(A)=H_{X}\left(a\right)+a{\bar{F}}_{X}\left(a\right). \end{array}$$ (8)

From (4) and using (6), (7) and (8), *dif*(*P*) is given by

$$\begin{array}{cc} dif(P) & =2\delta \left[H_{X}\left(a\right)+a{\bar{F}}_{X}\left(a\right)\right]\left[E\left(X\right)-H_{X}\left(a\right)-a{\bar{F}}_{X}\left(a\right)\right]\left[V\left(N\right)-E\left(N\right)\right]\\ & +2\delta E\left(N\right)a[E\left(x\right)-H_{X}\left(a\right)-a{\bar{F}}_{X}\left(a\right)]. \end{array}$$ (9)

As *dif*(*P*) is function of only one variable, in order to find the value of *a* that maximizes the difference, we substitute the gradient by the derivative with respect to *a*. The system (5) becomes

$$\begin{array}{c} E^{\prime }\left(AX\right)-E^{\prime }\left(A^{2}\right)=g\left(N\right)\left\{E^{\prime }\left(A\right)E\left(X\right)-{\left[E{\left(A\right)}^{2}\right]}^{\prime }\right\}. \end{array}$$ (10)

Differentiating (6), (7) and (8) with respect to *a*, we obtain,

$$\begin{array}{cc} E^{\prime }\left(AX\right) & =E\left(X\right)-H_{X}\left(a\right),\\ E^{\prime }\left(A^{2}\right) & =2a{\bar{F}}_{X}\left(a\right),\\ E^{\prime }\left(A\right) & ={\bar{F}}_{X}\left(a\right),\\ {\left(E{\left(A\right)}^{2}\right)}^{\prime } & =2{\bar{F}}_{X}\left(a\right)\left[H_{X}\left(a\right)+a{\bar{F}}_{X}\left(a\right)\right]. \end{array}$$

Then, using these last derivatives, (10) is

$$\begin{array}{c} E\left(X\right)-H_{X}\left(a\right)-2a{\bar{F}}_{X}\left(a\right)=g\left(N\right){\bar{F}}_{X}\left(a\right)\left[E\left(X\right)-2H_{X}\left(a\right)-2a{\bar{F}}_{X}\left(a\right)\right]. \end{array}$$ (11)

**Proposition 3**. *The function dif*(*P*) *attains a global maximum on D*.

*Proof*. The function *dif*(*P*) is a continuous and positive function in D = {*a*|*a* > 0}} and lim_*a* → ∞_ *dif*(*P*) = lim_*a* → 0_ *dif*(*P*)) = 0. Therefore, (11) has at least one solution and *dif*(*P*) attains a global maximum.

We consider two claim amount distributions: exponential and Pareto-Lomax.

*Exponential case:* If *X* ∼ exp(*γ*), *γ* > 0, the probability density function is given by *f*(*x*) = *γe*^ − *γx*^, then

$$\begin{array}{cc} H_{X}\left(a\right) & =\frac{1}{\gamma }-(\frac{1}{\gamma }+a)e^{-a\gamma }, \end{array}$$ (12)

$$\begin{array}{cc} {\bar{F}}_{X}\left(a\right) & =e^{-a\gamma }. \end{array}$$ (13)

Substituting the previous expressions in (9), *dif*(*P*) is

$$\begin{array}{c} dif\left(P\right)=\frac{2\delta }{\gamma }e^{-a\gamma }\{(1-e^{-a\gamma })(\frac{1}{\gamma }+2a)[V(N)-E(N)]+E\left(N\right)a\}. \end{array}$$

And, substituting (12) and (13) in (11), the equation that allows to obtain the value of *a* that maximizes *dif*(*P*) is

$$\begin{array}{c} 1-a\gamma =g\left(N\right)\left(2e^{-a\gamma }-1\right). \end{array}$$

The value of *a* that fulfills the previous expression, a*, depends on the relationship between *E*(*N*) and *V*(*N*). So, if *E*(*N*) > *V*(*N*), a* $\in (0,\frac{\ln2}{\gamma })\cup (\frac{1}{\gamma },\infty )$; if *E*(*N*) < *V*[*N*], $a^{*}\in (\frac{\ln2}{\gamma },\frac{1}{\gamma })$, and lastly, when *E*(*N*) = *V*(*N*), $a^{*}=\frac{1}{\gamma }$. If *N* is Poisson distributed with parameter *λ*, the optimal value for *a* coincides with the mean claim amount, the maximum difference is $\frac{2\delta E(N)}{\gamma^{2}e}$ and hence, for the insured, the best option is to purchase a deductible insurance with *a* = *E*(*X*) and a refundable insurance that covers *a*.

**Fig 1:**
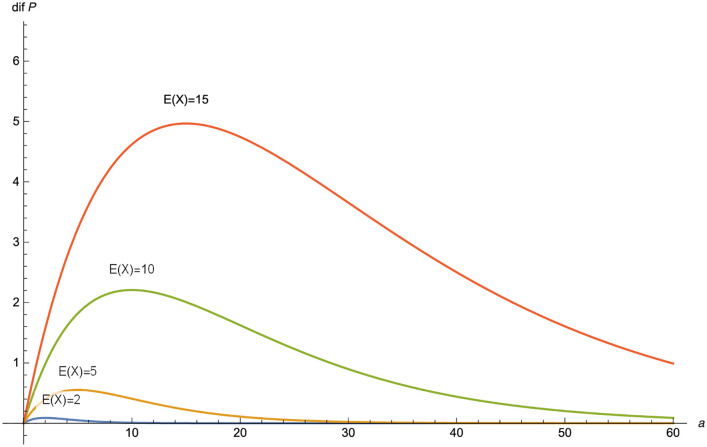
*dif*(*F*) as a function of *a* in the Poisson-exponential case for *δ* = 0.03 and *λ* = 1 for *E*(*X*) = 2, 5, 10, 15.

In Fig 1, we plot *dif*(*P*), in the Poisson-exponential case, as a function of *a* for different values of $E\left(X\right)=\frac{1}{\gamma }$.

*Pareto-Lomax case*: If *X* ∼ *Pareto*(*θ*, *ν*), the probability density function is given by *f*(*x*) = *θν*^*θ*^(*ν* + *x*)^ − *θ*−1^ and the mean is $E\left(X\right)=\frac{\nu }{\theta -1}$, *ν* > 0, *θ* > 2 (so that the variance is finite). Then,

$$\begin{array}{cc} H_{X}\left(a\right) & =\frac{1}{1-\theta }[\nu^{\theta }{\left(\nu +a\right)}^{1-\theta }-\nu ]-\frac{a\nu^{\theta }}{{\left(\nu +a\right)}^{\theta }},\\ {\bar{F}}_{X}\left(a\right) & ={\left(\frac{\nu }{\nu +a}\right)}^{\theta }. \end{array}$$

Substituting the previous expressions in (11)

$$\begin{array}{c} \nu +a\left(2-\theta \right)=g\left(N\right)\nu [2\nu^{\theta -1}{\left(\nu +a\right)}^{1-\theta }-1]. \end{array}$$

If *E*(*N*) > *V*(*N*), $a^{*}>\frac{\nu }{\theta -2}$, if *E*(*N*) = *V*(*N*) (Poisson case) $a^{*}=\frac{\nu }{\theta -2}$, and if *E*(*N*) < *V*(*N*), $a^{*}<\frac{\nu }{\theta -2}$.

Summarizing the results for the exponential and the Pareto-Lomax cases, in Table 2, the optimal values of *a* that maximizes *dif*(*P*) are included.

**Table 2:** Optimal *a* in absolute deductible in the Poisson-exponential and Poisson-Pareto-Lomax cases.

	*E*(*N*)<*V*(*N*)	*E*(*N*) = *V*(*N*)	*E*(*N*)>*V*(*N*)
Exp(*γ*)	$a^{*}\in (\frac{\ln2}{\gamma },\frac{1}{\gamma })$	$a^{*}=\frac{1}{\gamma }$	$a^{*}\in (0,\frac{\ln2}{\gamma })\cup (\frac{1}{\gamma },\infty )$
Pareto(*θ*,*ν*)	$a^{*}<\frac{\nu }{\theta -2}$	$a^{*}=\frac{\nu }{\theta -2}$	$a^{*}>\frac{\nu }{\theta -2}$

We now consider that *N* is Poisson distributed with parameter *λ*, and we do not specify the distribution of *X*. Now, *dif*(*P*) is linear on *δ* and depends on *a*, the only parameter of the deductible,

$$\begin{array}{c} dif\left(P\right)=\delta E\left(N\right)2a[E\left(X\right)-H_{X}\left(a\right)-a{\bar{F}}_{X}\left(a\right)]. \end{array}$$

The value of *a* that maximizes *dif*(*P*) is the solution to

$$\begin{array}{c} E\left(X\right)-H_{X}\left(a\right)-2a{\bar{F}}_{X}\left(a\right)=0, \end{array}$$

which can be rewritten as

$$\begin{array}{c} \int_{a}^{\infty }\left(x-2a\right)f\left(x\right)dx=0. \end{array}$$ (14)

The solution to (14) depends only on the distribution of *X*. Explicit expressions have been obtained in the previous two subsections for the exponential and the Pareto-Lomax distributions. For other claim amount distributions, only numerical solutions can be found. We calculate the optimal value of *a* and the maximum difference reached for several distributions of the claim amount. The parameters of the distributions are such that the expected value and the variance of the claim amount is the same, so the premium with a zero deductible insurance Π would be the same. The Pareto-Lomax distribution is not included in the comparison because there is no combination of *θ* and *ν* that fulfills *E*(*X*)^2^ = *V*(*X*), as in the exponential case. For the lognormal distribution, with parameters *μ* and *σ*, (14) is

$$\begin{array}{c} E\left(X\right)[1-\Phi (\frac{\ln a-(\mu +\sigma^{2})}{\sigma })]-2a[1-\Phi (\frac{\ln a-\mu }{\sigma })]=0, \end{array}$$ (15)

that, for the specific choice of parameters that fulfills *E*(*X*)^2^ = *V*(*X*), in order to compare with the exponential distribution, we obtain

$$\begin{array}{c} \begin{array}{cc} E(X) & \left\{1-\Phi \left[\frac{1}{\sqrt{\ln2}}\ln(\frac{a}{E(X)})-0.5\sqrt{\ln2}\right]\right\}\\ -2a & \{1-\Phi [\frac{1}{\sqrt{\ln2}}\ln(\frac{a}{E(X)})+0.5\sqrt{\ln2}]\}=0. \end{array} \end{array}$$

If the individual claim amount follows an Inverse-Gaussian distribution, *X* ∼ *IG*(*μ*, *λ*), *μ* > 0, *λ* > 0, the probability distribution function is given by $f\left(x\right)=\sqrt{\frac{\lambda }{2\pi x^{3}}}e^{\frac{-\lambda {\left(x-\mu \right)}^{2}}{2\mu^{2}x}}$, the expected value is *E*(*X*) = *μ* and its variance $V\left(X\right)=\frac{\mu^{3}}{\lambda }$. The combination of *μ* and *λ* that fulfills *E*(*X*)^2^ = *V*(*X*) is *μ* = *λ*. Then, the optimal value of *a* is obtained from

$$\begin{array}{c} \int_{a}^{\infty }\left(x-2a\right)\sqrt{\frac{\lambda }{2\pi x^{3}}}e^{\frac{-\lambda {\left(x-\mu \right)}^{2}}{2\mu^{2}x}}dx=0, \end{array}$$

equation that can be solved numerically.

In Table 3, the results of the optimal value a* and the maximum value of *dif*(*P*) depending on *E*(*N*) and *δ* are presented for an Exponential, a Lognormal and an Inverse-Gaussian distribution.

**Table 3:** Optimal value of *a* and difference (a*, *dif*(a*)) for *Exp*(*γ*), *Log*(*μ*, *σ*) and *IG*(*μ*, *λ*) claim amount distributions.

*E*(*X*)	*V*(*X*)	*Exp*(*γ*)	*Log*(*μ*, *σ*)	*IG*(*μ*, *λ*)
0.1	0.01	(0.1, 0.007*E*(*N*)*δ*)	(0.093, 0.001*E*(*N*)*δ*)	(0.102, 0.007*E*(*N*)*δ*)
0.5	0.25	(0.5, 0.184*E*(*N*)*δ*)	(0.468, 0.029*E*(*N*)*δ*)	(0.508, 0.168*E*(*N*)*δ*)
1	1	(1, 0.736*E*(*N*)*δ*)	(0.938, 0.332*E*(*N*)*δ*)	(1.016, 0.672*E*(*N*)*δ*)
2	4	(2, 2.943*E*(*N*)*δ*)	(1.877, 1.621*E*(*N*)*δ*)	(2.033, 2.69*E*(*N*)*δ*)
5	25	(5, 18.394*E*(*N*)*δ*)	(4.692, 9.902*E*(*N*)*δ*)	(5.082, 16.812*E*(*N*)*δ*)
10	100	(10, 73.576*E*(*N*)*δ*)	(9.388, 39.469*E*(*N*)*δ*)	(10.166, 67.248*E*(*N*)*δ*)

Table 3 shows that the maximum difference is proportional to the expected value of *N* and to the safety loading, which is the highest in the exponential case and the lowest in the Lognormal case. For each value of *E*(*X*), the optimal value of *a*, a*, is the lowest in the Lognormal case and the highest in the Inverse-Gaussian case.

### 3.2Proportional deductible

In this section, we focus our attention on the proportional deductible. We work with two types of proportional deductible: a first type in which the insured pays a percentage *α* of each claim, and, a second type in which we include a maximum loss for the insured, *B*. In a deductible with participation *α* ∈ (0, 1), *A* and *C* are defined in Table 4.

**Table 4:** Definition of *A* and *C* if a deductible with participation *α* ∈ (0, 1) is applied.

*X*	*A*	*C*
∀*X*	*αX*	(1 − *α*)*X*

In this case, *E*(*AX*) = *αE*(*X*^2^), *E*(*A*^2^) = *α*^2^ *E*(*X*^2^) and *E*(*A*) = *αE*(*X*), then

$$\begin{array}{c} dif\left(P\right)=2\delta \alpha \left(1-\alpha \right)\{E{\left(X\right)}^{2}\left[V\left(N\right)-E\left(N\right)\right]+E\left(N\right)E\left(X^{2}\right)\}, \end{array}$$

Using (5), the value *α* that maximizes the difference has to fulfill

$$\begin{array}{c} \left(1-2\alpha \right)[E\left(X^{2}\right)-g\left(N\right)E{\left(X\right)}^{2}]=0. \end{array}$$ (16)

**Proposition 4**. *The function dif*(*P*) *attains a global maximum in* $\alpha =\frac{1}{2}$.

*Proof*. The function *dif*(*P*) is a continuous and positive function in *D* = {*α*|*α* ∈ (0, 1)} and lim_*α* → 1_ *dif*(*P*) = lim_*α* → 0_ *dif*(*P*)) = 0. As the second factor in (16) is always different from 0, the critical point, $\alpha =\frac{1}{2}$, is a global maximum of *dif*(*P*).

Independently of the distribution of *N* and *X*, $\alpha =\frac{1}{2}$ is the value that maximizes the difference. Then, the maximum value of the difference is $\frac{\delta [E\left(N\right)V\left(X\right)+E{\left(X\right)}^{2}V\left(N\right)]}{2}$. If *N* ∼ *Pois*(*λ*), the maximum difference is $\frac{\delta \lambda E(X^{2})}{2}$.

Now, we generalize the proportional deductible including a maximum loss for the insured. In Table 5, a deductible with participation *α* ∈ (0, 1) and a maximum loss *B* > 0 is defined.

**Table 5:** Definition of *A* and *C* if a deductible with participation *α* ∈ (0, 1) with limit *B* > 0 is applied.

*X*	*A*	*C*
$X<\frac{B}{\alpha }$	*αX*	(1 − *α*)*X*
$X>\frac{B}{\alpha }$	*B*	*X* − *B*

If B tends to infinity, the first type of proportional deductible is obtained as a particular case.

If the deductible with participation *α* ∈ (0, 1) with limit *B* > 0 is applied,

$$\begin{array}{c} E(AX)=\alpha H_{X^{2}}(\frac{B}{\alpha })+B[E\left(X\right)-H_{X}(\frac{B}{\alpha })], \end{array}$$ (17)

$$\begin{array}{c} E(A^{2})=\alpha^{2}H_{X^{2}}(\frac{B}{\alpha })+B^{2}{\bar{F}}_{X}(\frac{B}{\alpha }), \end{array}$$ (18)

$$\begin{array}{c} E(A)=\alpha H_{X}(\frac{B}{\alpha })+B{\bar{F}}_{X}(\frac{B}{\alpha }). \end{array}$$ (19)

From (4), *dif*(*P*) can be rewritten as

$$\begin{array}{c} 2\delta \left[V\left(N\right)-E\left(N\right)\right][\alpha H_{X}(\frac{B}{\alpha })+B{\bar{F}}_{X}(\frac{B}{\alpha })][E\left(X\right)-\alpha H_{X}(\frac{B}{\alpha })-B{\bar{F}}_{X}(\frac{B}{\alpha })]\\ +2\delta E\left(N\right)\{\left(\alpha -\alpha^{2}\right)H_{X^{2}}(\frac{B}{\alpha })+B[E\left(X\right)-H_{X}(\frac{B}{\alpha })]-B^{2}{\bar{F}}_{X}(\frac{B}{\alpha })\} \end{array}$$

The deductible depends on two parameters, *α* and *B*. The partial derivatives with respect to *α* and *B*, from (17), (18) and (19), are

$$\begin{array}{c} \frac{\partial E(AX)}{\partial B}=E\left(X\right)-H_{X}(\frac{B}{\alpha }),\;\frac{\partial E(AX)}{\partial \alpha }=H_{X^{2}}(\frac{B}{\alpha }),\\ \frac{\partial E(A^{2})}{\partial B}=2B{\bar{F}}_{X}(\frac{B}{\alpha }),\;\frac{\partial E(A^{2})}{\partial \alpha }=2\alpha H_{X^{2}}(\frac{B}{\alpha }),\\ \frac{\partial E(A)}{\partial B}={\bar{F}}_{X}(\frac{B}{\alpha }),\;\frac{\partial E(A)}{\partial \alpha }=H_{X}(\frac{B}{\alpha }). \end{array}$$

Then, (5) is

$$\begin{array}{c} \begin{array}{c} E\left(X\right)-H_{X}(\frac{B}{\alpha })-2B{\bar{F}}_{X}(\frac{B}{\alpha })=g\left(N\right){\bar{F}}_{X}(\frac{B}{\alpha })\{E\left(X\right)-2[\alpha H_{X}(\frac{B}{\alpha })+B{\bar{F}}_{X}(\frac{B}{\alpha })]\},\\ \left(1-2\alpha )H_{X^{2}}(\frac{B}{\alpha })=g\left(N\right)H_{X}(\frac{B}{\alpha })\{E\left(X\right)-2[\alpha H_{X}(\frac{B}{\alpha })+B{\bar{F}}_{X}(\frac{B}{\alpha })]\right\} \end{array} \end{array}$$ (20)

The function *dif*(*P*) is a continuous and positive function in *D* = {(*α*, *B*)|*α* ∈ (0, 1), *B* > 0}. The critical points of the maximization problem are the solutions of the system of Eq (20). If we evaluate the function at the boundaries of *D*, it is easy to see that lim_*α* → 0_ *dif*(*P*) = lim_*B* → 0_ *dif*(*P*) = 0, lim_*α* → 1_ *dif*(*P*) equals to *dif*(*P*) in the absolute deductible case with parameter *a* = *B*, and lim_*B* → ∞_ *dif*(*P*) equals to *dif*(*P*) in the proportional deductible case. Therefore, we are not able to guarantee the existence of a global maximum for any *N* and *X*. Nevertheless, if *N* is Poisson distributed, we proof (see Proposition 5) that a global maximum does not exist. In this proposition, considering that the insured can choose only one of the parameters that define the deductible while the other is fixed by the insurer, we also obtain marginal maximums.

**Proposition 5**. *If N* ∼ *Pois*(*λ*), *there is no value of* (*B*, *α*) *that maximizes* (22), but marginal optimums exist. For a fixed *B*, $\alpha^{*}=\frac{1}{2}$ maximizes the difference. For a fixed $\alpha \leq \frac{1}{2}$ the maximum does not exist, and if $\alpha >\frac{1}{2}$ the maximum point, if it exists, fulfills $\int_{\frac{B}{\alpha }}^{\infty }\left(x-2B\right)f\left(x\right)dx=0$.

*Proof*. If *N* ∼ *Pois*(*λ*), *dif*(*P*) is

$$\begin{array}{c} dif\left(P\right)=2\delta E\left(N\right)\{(\alpha -\alpha^{2})H_{X^{2}}(\frac{B}{\alpha })+B[E\left(X\right)-H_{X}(\frac{B}{\alpha })]-B^{2}{\bar{F}}_{X}(\frac{B}{\alpha })\}, \end{array}$$ (21)

and (20) is

$$\begin{array}{c} \begin{array}{c} E\left(X\right)-H_{X}(\frac{B}{\alpha })-2B{\bar{F}}_{X}(\frac{B}{\alpha })=0,\\ \left(1-2\alpha \right)H_{X^{2}}(\frac{B}{\alpha })=0. \end{array} \end{array}$$ (22)

From the second equation in (22), knowing that $\int_{0}^{\frac{B}{\alpha }}x^{2}f\left(x\right)dx\neq 0$, we obtain $\alpha =\frac{1}{2}$. Substituting this value in the first equation, it is reduced to $\int_{2B}^{\infty }\left(x-2B\right)f\left(x\right)dx=0$, which is impossible because the integral is always different from 0. Then, there is no value of (*B*, *α*) that maximizes (22).

From $\frac{\partial dif(P)}{\partial \alpha }=0$ (the second equation of (22)), $\alpha =\frac{1}{2}$ is the critical point. As the sign of $\frac{\partial^{2}dif\left(P\right)}{\partial^{2}\alpha }$ at $\alpha =\frac{1}{2}$ is negative, the critical point is a maximum.

From $\frac{\partial dif(P)}{\partial B}=0$ (the first equation of (22)), the value of *B* that maximizes does not exist because $\int_{\frac{B}{\alpha }}^{\infty }\left(x-2B\right)f\left(x\right)dx$ is always different from 0 as $\frac{B}{\alpha }\geq 2B$, and if $\alpha >\frac{1}{2}$ the value of *B* that maximizes has to fulfill $\int_{\frac{B}{\alpha }}^{\infty }\left(x-2B\right)f\left(x\right)dx=0$.

**Fig 2:**
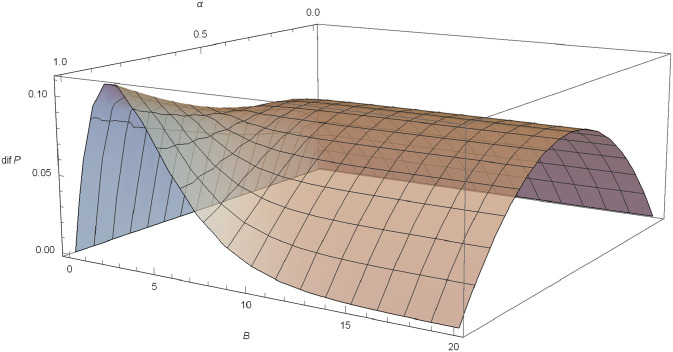
*dif*(*F*) in the Poisson-exponential case with *δ* = 0.03, *λ* = 1, *γ* = 0.6.

*Poisson-Exponential case*: If *N* ∼ *Pois*(*λ*) and *X* ∼ exp(*γ*), following Proposition 5, we know that there is no value of (*α*, *B*) that maximizes (21), but marginal optimums exist. For a fixed *B*, $\alpha^{*}=\frac{1}{2}$ maximizes the difference and for a fixed *α* > 0.5, the value $B^{*}=\frac{\alpha }{\gamma (2\alpha -1)}$ maximizes *dif*(*P*). For example, assuming *γ* = 0.6, *δ* = 0.03 and *λ* = 1, *dif*(*P*) as a function of *α* and *B* is plotted in Fig 2.

**Fig 3:**
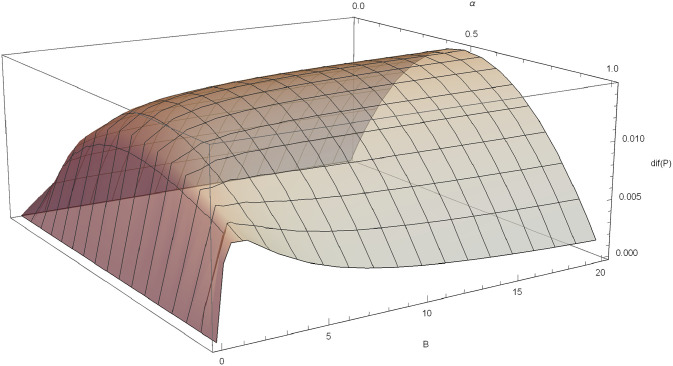
*dif*(*F*) in the Poisson-Pareto case with *δ* = 0.03, *λ* = 1, *θ* = 3.

*Poisson-Pareto case*: If *N* ∼ *Pois*(*λ*) and *c* for a fixed *B*, $\alpha^{*}=\frac{1}{2}$ maximizes *dif*(*P*) and for a fixed $\alpha >\frac{1}{2}$, $B^{*}=\frac{\alpha }{2\alpha (\theta -1)-\theta }$ also maximizes the difference. For example, if *θ* = 3, *δ* = 0.03 and *λ* = 1, *dif*(*P*) is plotted in Fig 3.

*Poisson-Lognormal case*: If *X* ∼ *LN*(*μ*, *σ*), for a fixed $\alpha >\frac{1}{2}$, the optimal value of *B* is the one that fulfils $\int_{\frac{B}{\alpha }}^{\infty }\left(x-2B\right)f\left(x\right)dx=0$, which can be written as,

$$\begin{array}{c} E\left(X\right)[1-\Phi (\frac{\ln\frac{B}{\alpha }-\left(\mu +\sigma^{2}\right)}{\sigma })]-2B[1-\Phi (\frac{\ln\frac{B}{\alpha }-\mu }{\sigma })]=0. \end{array}$$ (23)

In this case, it is not possible to obtain analytical results of the value *B* that optimizes the difference. Some numerical results are presented in Table 6 for different values of $\alpha >\frac{1}{2}$.

**Table 6:** Optimal *B* if the proportional deductible with limit is applied in the Poisson-Lognormal case for $\alpha >\frac{1}{2}$.

*α*	0.55	0.6	0.65	0.7	0.75	0.8	0.85	0.9	0.95	1
B*	11.79	3.17	2.01	1.59	1.38	1.26 1.18	1.128	1.088	1.058	

From Table 6, we see that B* decreases with *α* and if *α* = 1, we have in fact an absolute deductible and thus (23) is reduced to (15).

### 3.3 Mixture of absolute deductible and proportional deductible

In a mixture of absolute deductible with *a* > 0 and proportional deductible with *α* ∈ (0, 1), the insured pays the first *a* monetary units of each claim *X*, pays *a* if the claim amount is greater than *a* and less than $\frac{a}{\alpha }$, and if the claim amount is greater than $\frac{a}{\alpha }$ the proportional deductible is applied and the insured pays a percentage *α* of the claim amount. *A* and *C* are defined in Table 7.

**Table 7:** Definition of *A* and *C* if a mixture of absolute deductible *a* > 0 and proportional deductible *α* ∈ (0, 1) is applied.

*X*	*A*	*C*
*X* < *a*	*X*	0
$a<X<\frac{a}{\alpha }$	*a*	*X* − *a*
$X>\frac{a}{\alpha }$	*αX*	(1 − *α*)*X*

We define the different elements needed in the optimization of *dif*(*P*):

$$\begin{array}{cc} E(AX) & =H_{X^{2}}\left(a\right)+a[H_{X}(\frac{a}{\alpha })-H_{X}\left(a\right)]+\alpha [E\left(X^{2}\right)-H_{X^{2}}(\frac{a}{\alpha })],\\ E(A^{2}) & =H_{X^{2}}\left(a\right)+a^{2}[\bar{F}\left(a\right)-\bar{F}(\frac{a}{\alpha })]+\alpha^{2}[E\left(X^{2}\right)-H_{X^{2}}(\frac{a}{\alpha })],\\ E(A) & =H_{X}\left(a\right)+a[\bar{F}\left(a\right)-\bar{F}(\frac{a}{\alpha })]+\alpha [E\left(X\right)-H_{X}(\frac{a}{\alpha })]. \end{array}$$

This deductible depends on two parameters. We calculate the partial derivatives with respect to *α* and *a*,

$$\begin{array}{c} \frac{\partial E(AX)}{\partial a}=H_{X}(\frac{a}{\alpha })-H_{X}\left(a\right),\\ \frac{\partial E(A^{2})}{\partial a}=2a[\bar{F}\left(a\right)-\bar{F}(\frac{a}{\alpha })],\\ \frac{\partial E(A)}{\partial a}=\bar{F}\left(a\right)-\bar{F}(\frac{a}{\alpha }),\\ \frac{\partial E(AX)}{\partial \alpha }=E\left(X^{2}\right)-H_{X^{2}}(\frac{a}{\alpha }),\\ \frac{\partial E(A^{2})}{\partial \alpha }=2\alpha [E\left(X^{2}\right)-H_{X^{2}}(\frac{a}{\alpha })],\\ \frac{\partial E(A)}{\partial \alpha }=E\left(X\right)-H_{X}(\frac{a}{\alpha }). \end{array}$$

And the system of equations that allows us to obtain the critical points is,

$$\begin{array}{c} \begin{array}{c} H_{X}(\frac{a}{\alpha })-H_{X}\left(a\right)-2a[\bar{F}\left(a\right)-\bar{F}\left(\frac{a}{\alpha }\right)]=g\left(N\right)[\bar{F}\left(a\right)-\bar{F}\left(\frac{a}{\alpha }\right)][E(X)-2E(A)],\\ \left(1-2\alpha \right)[E\left(X^{2}\right)-H_{X^{2}}\left(\frac{a}{\alpha }\right)]=g\left(N\right)[E\left(X\right)-H_{X}\left(\frac{a}{\alpha }\right)][E(X)-2E(A)]. \end{array} \end{array}$$ (24)

The function *dif*(*P*) is a continuous and positive function in *D* = {(*α*, *a*)|*α* ∈ (0, 1), *a* > 0}. We evaluate *dif*(*P*) at the boundaries of *D*: lim_*α* → 1_ *dif*(*P*) = lim_*a* → ∞_ *dif*(*P*) = 0, lim_*α* → 0_ *dif*(*P*) equals to *dif*(*P*) in the absolute deductible case and lim_*a* → 0_ *dif*(*P*) equals to *dif*(*P*) in the proportional deductible case. As in the proportional deductible with limit *B*, this analysis does not allow us to assure the existence of a global maximum for any *N* and *X*. However, if *N* is Poisson distributed, we proof (see Proposition 5) that a global maximum does not exist and, additionally, we obtain marginal maximums.

**Proposition 6**. *If N* ∼ *Pois*(*λ*), *there is no value of* (*a*, *α*) *that maximizes* (25), but marginal optimums exist. For a fixed *a*, $\alpha^{*}=\frac{1}{2}$ maximizes the difference. For a fixed $\alpha \geq \frac{1}{2}$, the maximum does not exist, and if $\alpha <\frac{1}{2}$ the maximum point, if it exists, fulfills $\int_{a}^{\frac{a}{\alpha }}\left(x-2a\right)f\left(x\right)dx=0$.

*Proof*. If *N* ∼ *Pois*(*λ*),

$$\begin{array}{c} \begin{array}{cc} dif(P) & =2\delta E\left(N\right)\{\left(\alpha -\alpha^{2}\right)[E\left(X^{2}\right)-H_{X^{2}}(\frac{a}{\alpha })]+a[H_{X}(\frac{a}{\alpha })-H_{X}\left(a\right)]\}\\ & -2\delta E\left(N\right)\{a^{2}[\bar{F}\left(a\right)-\bar{F}(\frac{a}{\alpha })]\}, \end{array} \end{array}$$

and the system of Eq (24) is

$$\begin{array}{c} \begin{array}{c} H_{X}(\frac{a}{\alpha })-H_{X}\left(a\right)-2a[\bar{F}\left(a\right)-\bar{F}(\frac{a}{\alpha })]=0,\\ \left(1-2\alpha \right)[E\left(X^{2}\right)-H_{X^{2}}\left(\frac{a}{\alpha }\right)]=0, \end{array} \end{array}$$

or alternatively

$$\begin{array}{c} \begin{array}{c} \int_{a}^{\frac{a}{\alpha }}\left(x-2a\right)f\left(x\right)dx=0,\\ \left(1-2\alpha \right)\int_{\frac{a}{\alpha }}^{\infty }x^{2}f\left(x\right)dx=0. \end{array} \end{array}$$ (25)

From the second equation of (25), knowing that $\int_{\frac{a}{\alpha }}^{\infty }x^{2}f\left(x\right)dx\neq 0$, we obtain $\alpha^{*}=\frac{1}{2}$. Substituting this value in the first equation, we obtain $\int_{a}^{2a}\left(x-2a\right)f\left(x\right)dx=0$, which is impossible because the integral is always different from 0. Then, there is no value of (*a*, *α*) that maximizes (25).

From $\frac{\partial dif(P)}{\partial \alpha }=0$, $\alpha =\frac{1}{2}$ is the critical point. As the sign of $\frac{\partial^{2}dif\left(P\right)}{\partial^{2}\alpha }$ at $\alpha =\frac{1}{2}$ is negative, the critical point is a maximum.

From $\frac{\partial dif(P)}{\partial a}=0$, the first equation of (25), if $\alpha \geq \frac{1}{2}$, the value of *a* that maximizes does not exist because $\int_{a}^{\frac{a}{\alpha }}\left(x-2a\right)f\left(x\right)dx$ is always different from 0. If $\alpha <\frac{1}{2}$ the value of *a* that maximizes has to fulfill $\int_{a}^{\frac{a}{\alpha }}\left(x-2a\right)f\left(x\right)dx=0$.

*Poisson-Exponential case*: For *X* ∼ exp(*γ*), if $\alpha >\frac{1}{2}$, the value of *a* that optimizes the difference is obtained from the equation $\frac{2a-\frac{a}{\alpha }-\frac{1}{\gamma }}{\left(a-\frac{1}{\gamma }\right)}=e^{(\frac{1}{\alpha }-1)\gamma a}$ that has no explicit solution.

**Fig 4:**
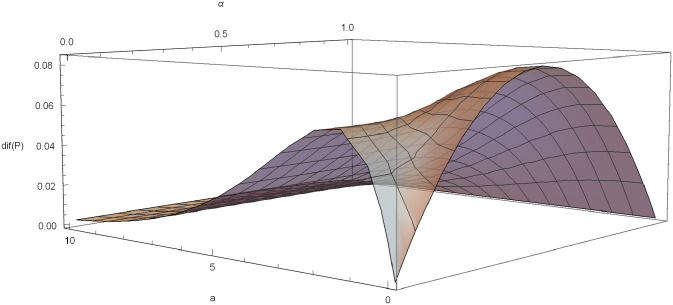
*dif*(*P*) in the Poisson-exponential case with *δ* = 0.03, *λ* = 1, *γ* = 0.6.

We can visualize graphically the behavior of *dif*(*P*) and its optimal values. For example, if *γ* = 0.6, *δ* = 0.03 and *λ* = 1, *dif*(*P*) is plotted in Fig 4.

**Fig 5:**
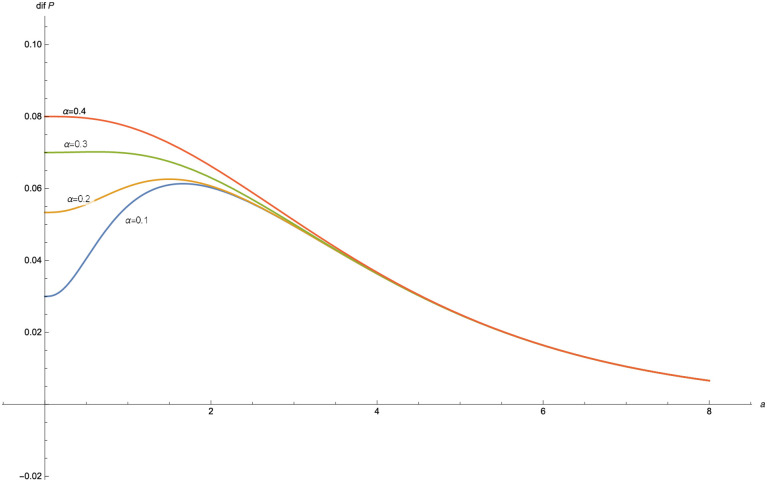
*dif*(*P*) for *α* = 0.1, 0.2, 0.3, 0.4, 0.5 in the Poisson-exponential case with *δ* = 0.03, *λ* = 1, *γ* = 0.6.

**Fig 6:**
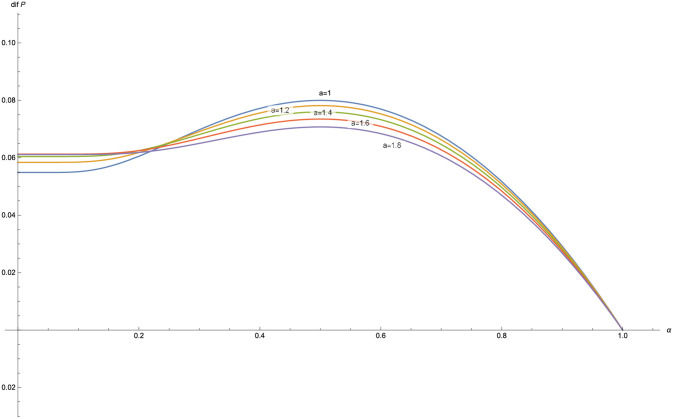
*dif*(*P*) for *a* = 1, 1.2, 1.4, 1.6, 1.8 in the Poisson-exponential case with *δ* = 0.03, *λ* = 1, *γ* = 0.6.

The function *dif*(*P*) is plotted for different values of *α*, with *δ* = 0.03, *λ* = 1 and *γ* = 0.6 (Fig 5) and for different values of *a* with *δ* = 0.03, *λ* = 1 and *γ* = 0.6 (Fig 6).

*Poisson-Pareto case*: If *X* ∼ *Pareto*(*θ*, 1), *θ* > 2, the value of *a* that optimizes the difference is obtained from the equation $\frac{2a(\gamma -1)-\gamma a-1}{2a\left(\gamma -1\right)-\frac{\gamma }{\alpha }a-1}={\left(\frac{\frac{a}{\alpha }+1}{a+1}\right)}^{-\theta }$.

**Fig 7:**
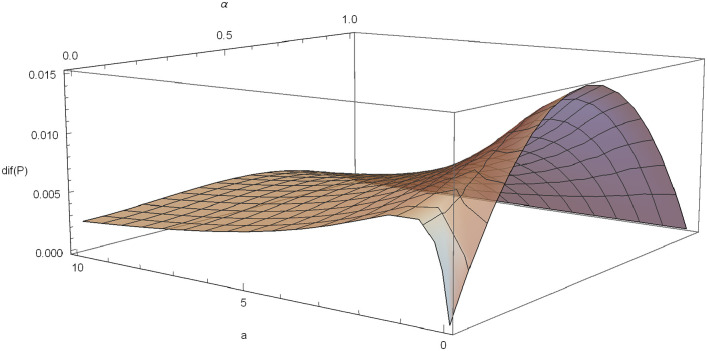
*dif*(*P*) in the Poisson-Pareto case with *δ* = 0.03, *λ* = 1, *θ* = 0.6.

In Fig 7, a graphical illustration of *dif*(*P*) is included for *θ* = 3, *δ* = 0.03 and *λ* = 1.

**Fig 8:**
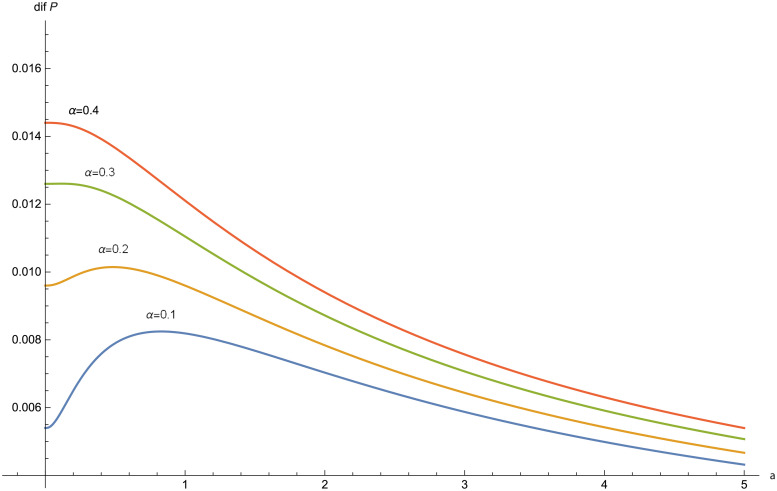
*dif*(*P*) for *α* = 0.1, 0.2, 0.3, 0.4 in the Poisson-Pareto case with *δ* = 0.03, *λ* = 1, *θ* = 3.

**Fig 9:**
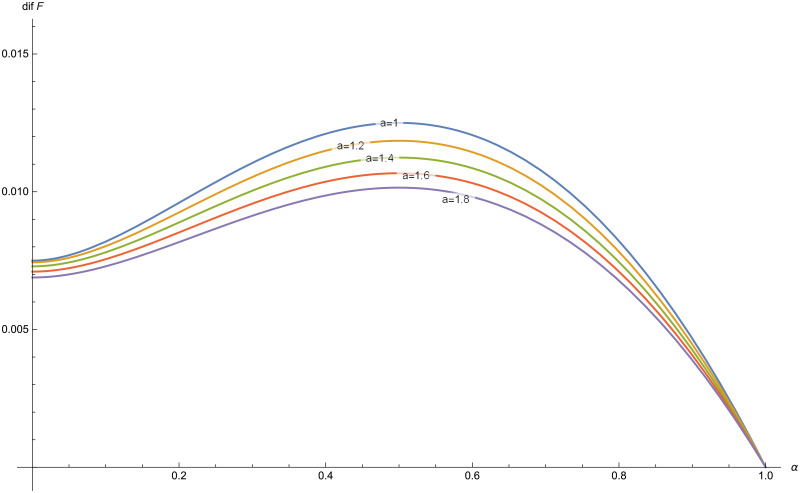
*dif*(*P*) for *a* = 1, 1.2, 1.4, 1.6, 1.8 in the Poisson-Pareto case with *δ* = 0.03, *λ* = 1, *θ* = 3.

Partial analysis of *dif*(*P*) with respect to *a* and *α* are plotted in Fig 8 and in Fig 9, respectively.

In the figures obtained for the exponential case (Figs 5 and 6) and for the Pareto case (Figs 8 and 9), we can observe a similar behavior in the optimization problem with respect to *a* and *α*.

*Poisson-Lognormal case*: If *X* ∼ *LN*(*μ*, *σ*), in order to obtain the optimal value of *a* that maximizes *dif*(*P*) for values of $\alpha \geq \frac{1}{2}$, the integral $\int_{a}^{\frac{a}{\alpha }}\left(x-2a\right)f\left(x\right)dx=0$ can be written as,

$$\begin{array}{ccc} E\left(X\right)[\Phi (\frac{\ln\frac{a}{\alpha }-\left(\mu +\sigma^{2}\right)}{\sigma })-\Phi (\frac{\ln a-(\mu +\sigma^{2})}{\sigma })]\\ -2a[\Phi (\frac{\ln\frac{a}{\alpha }-\mu }{\sigma })-\Phi (\frac{\ln a-\mu }{\sigma })] & = & 0. \end{array}$$

Due to the impossibility of analytically solving the previous equation, Table 8 shows some numerical results from which we see that a* decreases with *α*.

**Table 8:** Optimal value of *a* in the Poisson-Lognormal case for $\alpha <\frac{1}{2}$.

*α*	0.05	0.1	0.15	0.2	0.25	0.3	0.35	0.4	0.45
a*	1.053	1.052	1.042	1.007	0.934	0.81	0.625	0.377	0.096

### 3.4 All-nothing deductible

In this section, we analyze the all-nothing deductible with participation *M* > 0. The idea is that if the individual claim amount, *X*, is less than *M*, the insurer pays the whole claim, but if *X* is greater than *M*, the insurer does not pay anything (see [15]).

In Table 9, *A* and *C* are defined.

**Table 9:** All-nothing deductible with participation *M*.

*X*	*A*	*C*
*X* < *M*	0	*X*
*X* > *M*	*X*	0

This deductible does not fulfill Proposition 1 because *A* and *C* are not commonotic risks. Then, the following results are not focused on obtaining the optimal deductible for the insured because it is not possible to maintain that the function *dif*(*P*) is always positive.

If the all-nothing deductible is applied, *E*(*AX*) = *E*(*A*^2^) = *E*(*X*^2^) − *H*_*X*^2^_(*M*), and *E*(*A*) = *E*(*X*) − *H*_*X*_(*M*). Hence, the expression for *dif*(*P*) is

$$\begin{array}{c} dif\left(P\right)=2\delta \left[E\left(X\right)-H_{X}\left(M\right)\right]H_{X}\left(M\right)[V(N)-E(N)]. \end{array}$$ (26)

If *N* ∼ *Pois*(*λ*), then *dif*(*P*) = 0 regardless of the claim amount distribution. Then, in the Poisson case, for the insured it is the same to purchase a Zero Deductible Insurance policy or a Deductible Insurance policy together with a Refundable Deductible Insurance policy.

Knowing that *E*(*X*) − *H*_*X*_(*M*)>0, we can observe in (26) that the sign of *dif*(*P*) depends on the sign of *V*(*N*) − *E*(*N*). Then, *dif*(*P*) can be positive or negative. The explanation is that, in this deductible, *A* and *C* are not commonotic risks (see [21]), and therefore the initial hypothesis of this paper is not fulfilled. That is to say, in this deductible, *Cov*(*A*, *C*) is not a positive value. In fact, knowing that *E*(*AC*) = *E*(*AX*) − *E*(*A*^2^) = 0, *Cov*(*A*, *C*) = −*E*(*A*)*E*(*C*)<0. From (3), the positiveness of *Cov*(*A*, *C*) is a sufficient, but not necessary, condition for the positiveness of *dif*(*P*). We are only interested in the situations in which *dif*(*F*) is positive, therefore we impose that *V*(*N*)>*E*(*N*).

In order to obtain the value that maximizes *dif*(*P*), we need the following two previous derivatives,

$$\begin{array}{c} E^{\prime }\left(AX\right)=E^{\prime }\left(A^{2}\right)=-M^{2}f\left(M\right),\\ E^{\prime }\left(A\right)=-Mf\left(M\right), \end{array}$$

and from (5), the first order condition is,

$$\begin{array}{c} E\left(X\right)=2H_{X}\left(M\right). \end{array}$$ (27)

Considering that *H*_*X*_(*M*) is a continuous and increasing function in *D* = {*M*|*M* > 0}, with *H*_*X*_(0) = 0 and lim_*M* → ∞_ *H*_*X*_ = *E*(*X*), we can assure the existence of a critical point. It is easy to see that *dif*′′(*P*) is always negative, then the critical point is a maximum.

In the exponential case, (27) is (2*γM* + 2)*e*^ − *Mγ*^ = 1 and for the *Pareto*(*θ*, *ν*), it is

$$\begin{array}{c} {\left(\frac{\nu }{\nu +M}\right)}^{\theta }=\frac{\nu }{2(\nu +M\theta )}. \end{array}$$ (28)

In Table 10, some numerical results for the exponential distribution are obtained.

**Table 10:** Optimal value of M in the exponential case.

*E*(*X*)	1	2	3	4	5	6	10	20
*M*	1.6783	3.3567	5.035	6.7134	8.3917	10.07	16.783	33.567

If *X* follows a *Pareto*(*θ*, *ν*) distribution, the optimal values of *M* are obtained solving numerically (28). The results are shown in Table 11.

**Table 11:** Optimal value of M in the Pareto case.

*ν* *θ*	2.1	2.5	3	3.5	4	5
1	2.1189	1.4176	1	0.771650	0.627942	0.457323
1.5	3.17833	2.12645	1.5	1.15747	0.941913	0.685984
2	4.23778	2.83527	2	1.5433	1.2559	0.914645
2.5	5.29722	3.54409	2.5	1.92912	1.5698	1.14331

If *X* ∼ *LN*(*μ*, *σ*), then (27) is,

$$\begin{array}{c} \Phi (\frac{\ln M-(\mu +\sigma^{2})}{\sigma })=\frac{1}{2}, \end{array}$$

that implies ln *M* = (*μ* + *σ*^2^), hence the value of M that maximizes *dif*(*P*) is *M* = *e*^(*μ* + *σ*^2^)^.

## 4 Conclusions

In this paper, we present a theoretical framework to analyze the advantage that the insured can obtain by purchasing a Refundable Deductible Insurance policy. Several principles can be applied by the insurer to calculate premiums: if the mean principle is applied, this advantage is null, whereas with the variance principle, the commonotonicity of the parts of the claim covered by the insurer and the insured guarantees the advantage.

The deductible parameters that maximize the advantage obtained by the insured depend on the expected value and variance of the number of claims and the distribution of the individual claim amount. We conclude that the existence and the value of the maximums depend on the type of deductible applied. We proof the existence of a global maximum if an absolute deductible or a proportional deductible are applied. In the proportional deductible case, we find that if the insured chooses to pay the fifty per cent of each claim, the advantage obtained by purchasing a Refundable Deductible Insurance policy is the maximum one. In the absolute deductible case, explicit expressions of the part of the claim paid by the insured that maximizes the advantage are obtained for several distributions of the claim amount and the number of claims. In the other cases (proportional deductible with limit and mixture of absolute and proportional deductible), if the number of claims is Poisson distributed, we proof that the global maximum does not exist.

The results of the paper can help the insured in his/her decision-making process regarding risk coverage.
